# Role of pirenoxine in the effects of catalin on in vitro ultraviolet-induced lens protein turbidity and selenite-induced cataractogenesis in vivo

**Published:** 2011-07-12

**Authors:** Chao-Chien Hu, Jiahn-Haur Liao, Kuang-Yang Hsu, I-Lin Lin, Ming-Hsuan Tsai, Wen-Hsin Wu, Tzu-Tang Wei, Yi-Shiang Huang, Shih-Jiuan Chiu, Hsiang-Yin Chen, Shih-Hsiung Wu, Tzu-Hua Wu

**Affiliations:** 1 Department of Ophthalmology, Shin Kong Wu Ho-Su Memorial Hospital, Taipei, Taiwan; 2 School of Medicine, Fu-Jen Catholic University, Hsinchuang, Taiwan; 3 School of Medicine, Taipei Medical University, Taipei, Taiwan; 4 Institute of Biological Chemistry, Academia Sinica, Taipei, Taiwan; 5 School of Pharmacy, College of Pharmacy, Taipei Medical University, Taipei, Taiwan

## Abstract

**Purpose:**

In this study, we investigated the biochemical pharmacology of pirenoxine (PRX) and catalin under in vitro selenite/calcium- and ultraviolet (UV)-induced lens protein turbidity challenges. The systemic effects of catalin were determined using a selenite-induced cataractogenesis rat model.

**Methods:**

In vitro cataractogenesis assay systems (including UVB/C photo-oxidation of lens crystallins, calpain-induced proteolysis, and selenite/calcium-induced turbidity of lens crystallin solutions) were used to screen the activity of PRX and catalin eye drop solutions. Turbidity was identified as the optical density measured using spectroscopy at 405 nm. We also determined the in vivo effects of catalin on cataract severity in a selenite-induced cataract rat model. Sodium dodecyl sulfate polyacrylamide gel electrophoresis (SDS–PAGE) was applied to analyze the integrity of crystallin samples.

**Results:**

PRX at 1,000 μM significantly delayed UVC-induced turbidity formation compared to controls after 4 h of UVC exposure (p<0.05), but not in groups incubated with PRX concentrations of <1,000 μM. Results were further confirmed by SDS–PAGE. The absolute γ-crystallin turbidity induced by 4 h of UVC exposure was ameliorated in the presence of catalin equivalent to 1~100 μM PRX in a concentration-dependent manner. Samples with catalin-formulated vehicle only (CataV) and those containing PRX equivalent to 100 μM had a similar protective effect after 4 h of UVC exposure compared to the controls (p<0.05). PRX at 0.03, 0.1, and 0.3 μM significantly delayed 10 mM selenite- and calcium-induced turbidity formation compared to controls on days 0~4 (p<0.05). Catalin (equivalent to 32, 80, and 100 μM PRX) had an initial protective effect against selenite-induced lens protein turbidity on day 1 (p<0.05). Subcutaneous pretreatment with catalin (5 mg/kg) also statistically decreased the mean cataract scores in selenite-induced cataract rats on post-induction day 3 compared to the controls (1.3±0.2 versus 2.4±0.4; p<0.05). However, catalin (equivalent to up to 100 μM PRX) did not inhibit calpain-induced proteolysis activated by calcium, and neither did 100 μM PRX.

**Conclusions:**

PRX at micromolar levels ameliorated selenite- and calcium-induced lens protein turbidity but required millimolar levels to protect against UVC irradiation. The observed inhibition of UVC-induced turbidity of lens crystallins by catalin at micromolar concentrations may have been a result of the catalin-formulated vehicle. Transient protection by catalin against selenite-induced turbidity of crystallin solutions in vitro was supported by the ameliorated cataract scores in the early stage of cataractogenesis in vivo by subcutaneously administered catalin. PRX could not inhibit calpain-induced proteolysis activated by calcium or catalin itself, and may be detrimental to crystallins under UVB exposure. Further studies on formulation modifications of catalin and recommended doses of PRX to optimize clinical efficacy by cataract type are warranted.

## Introduction

Cataracts have been a significant global problem of visual impairment for centuries. It is estimated that the number of cataract victims will increase by about one-third over the next 20 years, and that cataracts will remain the leading cause of visual impairment in all regions of the world [1,2]. Without intervention, the global number of individuals with cataracts and other eye disorders will increase from 44 million in 2000 to 76 million in 2020 [3]. Although cataracts can be surgically cured, in some countries the treatment is unavailable. Cataract prevention is, therefore, still important, and the discovery of effective anti-cataract drugs is an important issue in eye health.

Pirenoxine (PRX), a pyridophenoxazine compound resembling xanthommatin, competitively inhibits the sulfhydryl combination of quinoid substances with lens proteins. Ogino et al. [4,5] proposed that the histopathology of cataracts was caused by various quinoid substances. PRX was first introduced in Japan in 1958 to prevent early cataracts, and it is still widely used in Taiwan. Although PRX has been used clinically for many years [6-8], scientific evidence of its efficacy is lacking. A 2006 paper criticized doctors for prescribing PRX without such proof [9].

The effectiveness of preparations for cataracts, particularly PRX, should be verified before ophthalmologists further prescribe them. Although some PRX studies have shown beneficial effects, poor design (e.g., non-double blinded, insufficient case numbers, and short follow-up periods) has made the conclusions questionable [10]. Evidence-based studies are still needed to clarify the biochemical and clinical roles of PRX in preventing cataract formation.

Risk factors for cataract formation include eye inflammation, environmental hazards, drug-induced side-effects, age, gender and genetic factors. Among the many risk factors associated with cataract formation, it has been determined that various ions may play roles in cataractogenesis [11,12]. Our previous study established a novel in vitro selenite ion-induced lens protein precipitation system [13]. Selenite and calcium at the millimolar (mM) level have been demonstrated to cause lens crystallin aggregation [13-15]. The ability of selenite to cause cataracts in an animal model was first described in 1977 [16]. Selenite’s involvement in cataract generation is related to raised levels of calcium resulting from the increased lens membrane permeability occurring naturally in older patients.

The results of tests focusing on accumulated ^75^Se in soluble and insoluble proteins of the lens [17] and the development of cortical opacities in a medium containing selenite [18] indicate that selenite enters the lens itself. The accumulation of selenite in the lens may cause cataract formation and explains the high selenium content [19,20] in human lenses with increased opacity. Opaque lenses also show an accumulation of calcium [21,22]. An increase in intracellular Ca^2+^ triggers calcium-activated enzymes such as calpains. The incubation of soluble proteins from rat lens with calpain II protease caused precipitation of β-crystallin polypeptides, which then cleaved at their similar NH_2_-terminal extensions [23]. Another study also revealed that the addition of the calpain II inhibitor, E64, prevented calpain-induced crystallin proteolysis [24].

The human lens absorbs both ultraviolet A (UVA, 320~400 nm) and ultraviolet B (UVB, 290~320 nm) rays and transmits the visible spectrum. A positive correlation between the prevalence of senile cataracts and levels of climatic UV radiation was reported earlier [25], and the decreased lens clarity may result from the high-molecular-weight disulfide-linked proteins aggregating inside the aging human lens [26]. Oxidative stress also modifies protein disulfide formation in the human lens [27]; for example, the observed increased production of reactive oxygen species (ROS), decreased glutathione level, and increased disulfide bonds and carbonyl content in lenses further result in cross-linkage and insoluble large-molecular protein precipitants, causing light scattering after exposure to UVB [28]. Absorption of UVB by lens molecules was also reported to lead to photo-oxidative stress [29] and increased risk of cortical and subcapsular cataracts [25,30]. Exposure to ultraviolet C (UVC, 200~290 nm) changed the secondary structure and decreased the chaperone activities of α-crystallins [31,32], resulting in increased turbidity and free radicals [33].

PRX with selenite/calcium ditopic complexation [14] provides a rationale for using PRX-based catalin eye drops in eye clinics. However, the role of PRX in catalin's effects on selenite-, calcium-, and UVB-induced formation of crosslinked proteins and UVC overexposure-induced cataract formation is still unknown. Therefore, to determine the role of pure PRX in the activities of catalin eye drops and formulation effects, this study employed in vitro cataractogenesis assay systems of selenite/calcium-induced precipitation, UVB/C photo-oxidation of lens crystallins, and calpain-induced proteolysis to investigate the mechanisms of the drug. The in vivo efficacy of catalin was then confirmed in a selenite-induced cataractogenesis rat model.

## Methods

### General

Porcine lenses were decapsulated and homogenized in lens buffer containing 50 mM Tris-HCl, 0.1 M NaCl, 5 mM EDTA, 0.01% β-mercaptoethanol, and 0.02% sodium azide, at pH 8.0. After centrifugation at 16,060× g for 30 min, the supernatant was collected, and the protein concentration determined according to the Bradford method (BioRad Laboratories, Irvine, CA). Pure PRX was purchased from Hangzhou Dayangchem (Hangzhou, Zhejiang, China), and catalin was from Kaken Pharmaceutical (Tokyo, Japan). To determine the dosing efficacy of the catalin eye drop solution against cataract formation induced by various pathological factors, catalin solutions were used and prepared according to instructions, with one exception; the volume of the vehicle solvent used was minimized to conform to the study design. PRX doses (16~100 μM) of catalin product powder for the in vitro assay were calculated according to the labeled PRX content of catalin and to clinically recommended doses for humans. Samples for the turbidity assay were incubated at 37 °C using microtiter plates (Microtest^TM^ 96; Falcon; Fisher Scientific, Franklin Lakes, NJ), and turbidity was measured as the optical density (OD) at 405 nm, using an enzyme-linked immunosorbent assay (ELISA) reader (Tecan Sunrise^TM^; Tecan Group, Männedorf, Switzerland).

### Photo-oxidation of γ-crystallins by UVB or UVC in vitro

Chemical modifications or oxidation of lens proteins by UV irradiation may also contribute to opacities and were shown to cause the turbidity of γ-crystallin [34,35]. In this anti-UVB assay, 200 µl of incubation lens buffer mixtures containing 20 mg/ml γ-crystallin, with or without various concentrations of PRX (0, 0.1, 1, 10, or 100 μM) were exposed to UVB (Vilber Loumat bio-link crosslinker; 312 nm) for 0, 2, 4, and 6 h, as described by Andley et al. [36]. In the anti-UVC assays, 200 µl of incubation lens buffer mixtures containing 0.4 mg/ml γ-crystallin, with or without various concentrations of PRX (0, 10^−3^, 10^−2^, 0.1, 0.3, and 1 mM), or catalin eye drops equivalent to up to 100 μM PRX, were exposed to UVC (UVP CL-1000; 254 nm) for 0, 1, 2, 3, and 4 h. After the measurement, the mixture from each well was collected for a sodium dodecylsulfate PAGE (SDS–PAGE) analysis.

### m-Calpain-induced proteolysis of crystallins

To determine the in vitro exogenous calpain-induced lens protein turbidity, exogenous lens calpain was activated by adding 1.5 mM calcium to induce exogenous calpain II, as modified from the Shih et al. [37] study. Incubations at 200 µl were prepared by adding 5 mg of lens-soluble proteins/ml, 7.5 units calpain II (from porcine kidney; Calbiochem, Torrey Pines, CA) with 100 μM PRX, various concentrations of catalin, (16, 32, 80, and 100 μM), or catalin-formulated vehicle only (CataV), while 3 mM EDTA or 100 μM E64 was used as the positive control. Proteolysis was initiated by adding 1.5 mM Ca^2+^, processed for 10 min at 37 °C, and was terminated by the addition of EDTA to a final concentration of 3 mM. Protein patterns were then analyzed by SDS–PAGE.

### In vitro lens crystallin turbidity assays induced by 10 mM selenite or calcium

The crystallin turbidity assays were performed by adding sodium selenite [13] or calcium ions [38] to cause lens turbidity. The incubation lens buffer mixture at 200 µl contained 50 mg/ml of lens-soluble proteins, with or without various concentrations of PRX (0, 0.03, 0.1, 0.3, and 1 μM) or a catalin eye drop solution containing PRX equivalent to 0, 0.03, 0.1, 0.3, and 1 μM. Selenite or calcium at 10 mM was then added to induce turbidity. The absolute OD and changes in the OD were calculated for each incubation day and treatment.

### Selenite-induced cataract animal model

The ability of selenite to cause cataracts in an animal model was first described in 1977 [16]. Sprague-Dawley rat pups, aged 9 days, were obtained from The National Science Council Animal Center (Taipei, Taiwan) and housed with their mothers under standard conditions. All experiments were performed in accordance with the Institute for Laboratory Animal Research Guide for the Care and Use of Laboratory Animals. The rat pups were given normal rat chow and water ad libitum. Beginning from postnatal day 11, rat pups were divided into four groups (n=3~7) and administered subcutaneous catalin solutions (0, 2.5, or 5 mg/kg) or the vehicle for 3 days. On postnatal day 14, all pups were given a single injection of 19 μmol/kg sodium selenite (pH 9.0) subcutaneously except those with no treatment (normal controls). To minimize bias, the transparency status of all eyes was observed daily by an ophthalmologist, who was blind to the treatment protocols, using slip lamp to score on a 4-point scale: 0 (extremely clear), 1 (diffuse scattered nuclear opacities), 2 (formation of pinpoint cataracts), and 3 (mature dense opacities involving the entire lens) [39]. Mean transparency scores were calculated for each group, and the lenses were then collected at the end of the experiment. Lens proteins were extracted and centrifuged at 15,000× g and 4 °C for 15 min to obtain the supernatant and insoluble pellets for the SDS–PAGE analysis.

### Statistical analysis

Average turbidity formation in absolute OD, changes from the baseline or percentage changes versus the controls. The cataract scores after various treatments, are expressed as the mean±standard error of the mean (n≥3). The scores were statistically analyzed using an unpaired *t*-test between two groups, or by Kruskal–Wallis one-way ANOVA on ranks with post-hoc Dunn’s test, using SigmaStat 2.03 (Systat Software, San Jose, CA). Differences were considered significant when the p value was <0.05.

## Results

### Effects of PRX and catalin on UVB- and UVC-induced photo-oxidation of γ-crystallins

UV irradiation is a significant risk factor for cataractogenesis. After 6 h of UVB exposure, none of the concentrations of PRX (0.1, 1, 10, or 100 μM) inhibited or delayed turbidity formation. In fact, turbidity in the PRX groups was even higher than in the controls. However, a high concentration of PRX (1 mM) inhibited turbidity formation induced by 4 h of UVC exposure. There was a significant statistical difference in the OD changes between the control and PRX groups (0.489±0.007 versus 0.401±0.015, p<0.05). These results showed that at a high concentration (1 mM), PRX may protect γ-crystallin from continuous insults by UVC. Therefore, PRX delayed turbidity formation induced by UVC but not by UVB (data not shown).

**Figure 1:**
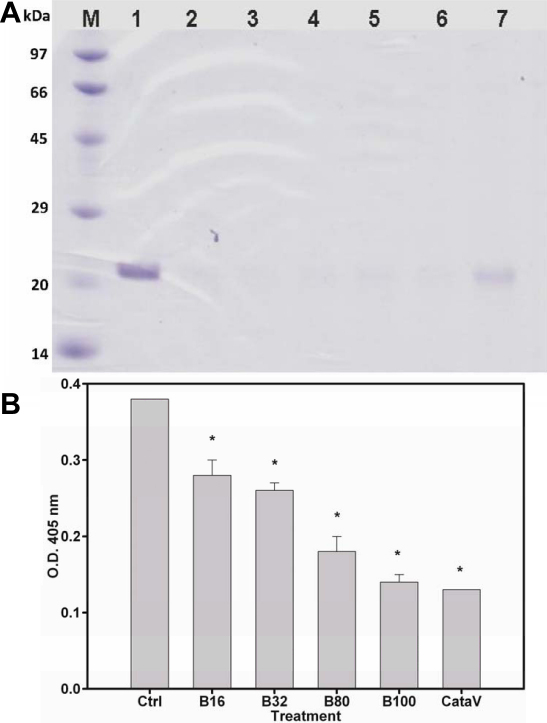
Photo-protection against 4 h of ultraviolet C (UVC) exposure by pirenoxine (PRX) and catalin. **A**: A 12% SDS–PAGE of γ-crystallins incubated with or without PRX (0~1000 μM). M, Bioladder^®^ protein marker; lane 1, normal; 2, control; 3, 1 μM PRX; 4, 10 μM PRX; 5, 100 μM PRX; 6, 300 μM PRX; 7, 1000 μM PRX. The protein bands in Lane 7 was similar to lane 1 (normal). **B**: Turbidity expressed as optical density (OD) was determined by spectroscopy at 405 nm. The y-axis indicates the OD of γ-crystallins incubated with various doses of catalin. B16: catalin containing 16 μM PRX, B32: catalin equivalent to 32 μM PRX, B80: catalin equivalent to 80 μM PRX, B100: catalin equivalent to 100 μM PRX, CataV: catalin-formulated vehicle only. *p<0.05 versus control (Ctrl) group.

Patterns of γ-crystallin in the control group showed that protein expression significantly decreased at 21 kDa. These results demonstrate that γ-crystallin was degraded by UVC exposure (Figure 1A). Patterns of γ-crystallin in groups with higher concentrations of PRX were more similar to normal ones. Similar results were also observed for catalin solutions (Figure 1B).

Catalin containing PRX equivalents of 16~100 μM significantly ameliorated the turbidity of lens crystallins following 4 h of UVC exposure in a concentration-dependent manner compared to the controls (p<0.05). Samples containing only catalin formulated vehicle had similar potencies of anti-UVC activity to those containing PRX equivalent to 100 μM.

### PRX or catalin failed to inhibit m-calpain-induced proteolysis of crystallins

**Figure 2:**
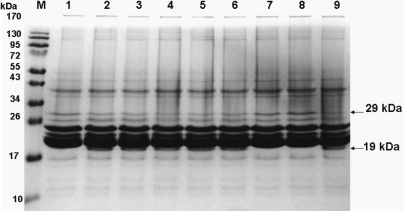
Effects of various treatments on calpain-induced lens proteolysis. A 12% SDS–PAGE analysis shows that proteolyzed proteins were seen at 19 kDa (lane 2–6, lane 9) while EDTA and calpain inhibitor E64 inhibited proteolysis. M, Fermentas prestained protein ladder^®^ marker; lane 1, normal; 2, control; 3, 100 μM PRX; 4, catalin equivalent to 100 μM PRX; 5, catalin equivalent to 80 μM PRX; 6, catalin equivalent to 32 μM PRX; 7, 3 mM EDTA; 8, 100 μM E64; 9, catalin-formulated vehicle only.

SDS–PAGE (Figure 2) of the control group showed there was a decrease in 29-kDa proteins and an increase in 19-kDa proteins compared to normal samples, which indicated that β- and α-crystallins were degraded by exogenous calpain II, which was activated by calcium in this assay system. Figure 2 shows that presence of catalin or PRX changed the protein patterns in SDS–PAGE, since no difference was found in the control with the PRX (100 μM) or catalin solution-treated groups (containing 0, 32, 80, and 100 μM PRX), while no truncated 19-kDa proteins appeared in the positive control groups (EDTA, E64).

### Effect of PRX against 10 mM selenite- and calcium-induced lens protein turbidity

**Figure 3:**
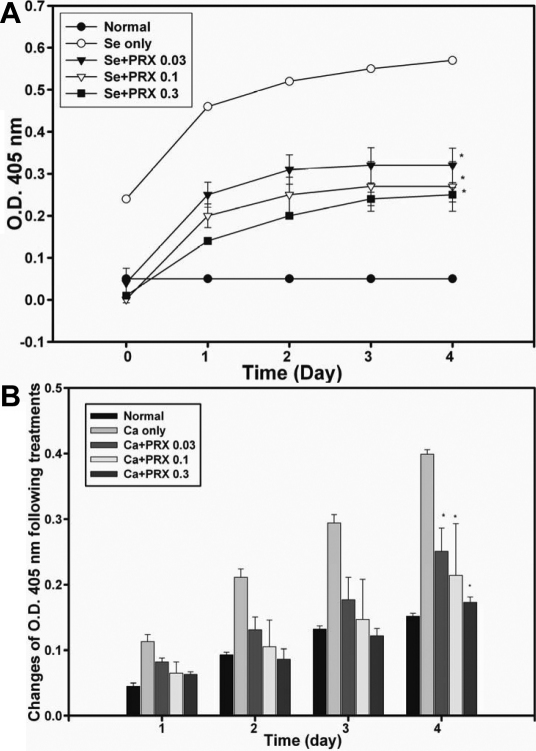
Pirenoxine (PRX) ameliorated selenite- and calcium-induced lens protein turbidity. Turbidity was expressed as an optical density (OD) and was determined by spectroscopy at 405 nm for 4 days. *0.03, 0.1, and 0.3 μM PRX versus controls (selenite or calcium only) at p<0.05. **A**: Selenite-induced turbidity formation in terms of the absolute OD was plotted versus incubation days. **B**: Calcium-induced turbidity formation expressed as absolute changes in lens turbidity in terms of the OD from the baseline were plotted by incubation day.

The OD of the selenite control group increased to 0.57 while those for the 0.03, 0.1, and 0.3 μM PRX treatment groups exhibited significantly smaller increases as shown in Figure 3A (p<0.05). In the calcium-induced turbidity assays on day 4, the absolute changes in OD from day 0 for the control and various concentrations of PRX (0.03, 0.1, and 0.3 μM) groups are shown in Figure 3B, and all PRX groups had statistically significantly smaller turbidity changes compared to the controls (p<0.05). Various concentrations of PRX (0.03~0.3 μM) inhibited turbidity formation in a dose-dependent manner against selenite- and calcium-induced turbidity of lens crystallins.

### Catalin delayed selenite-induced turbidity formation of crystallin solutions in vitro and in a cataract model of rats

**Figure 4:**
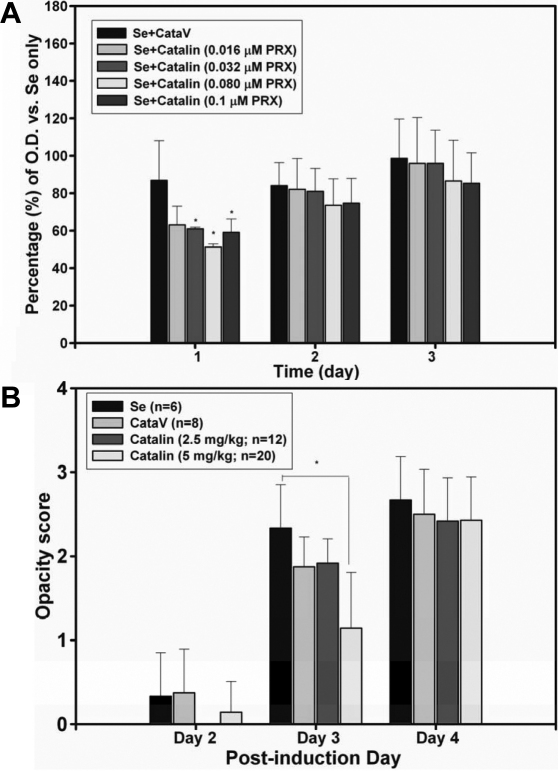
Effect of catalin on selenite-induced lens turbidity and cataract scores. **A**: Percentages of the optical density (OD) for lens turbidity of the selenite -only group influenced by the presence of catalin equivalent to 0.016–0.1 μM PRX or the catalin formulated vehicle (CataV) were compared. Turbidity was determined by spectroscopy at 405 nm. *catalin (0.032–0.1 μM PRX) versus CataV at p<0.05. **B**: Average cataract scores of groups treated with various doses of catalin in a selenite-induced cataract rat model. Rats (n=6~20) pretreated with catalin solutions (0, 2.5, and 5 mg/kg) or the CataV administered subcutaneously for 4 days. Each lens cataract score was classified into four degrees (0–3) from clear to mature dense. The average cataract score of each group is expressed as the mean±standard error and is plotted versus the post cataract induction day. *catalin (5 mg/kg) versus Se controls at p<0.05.

**Figure 5:**
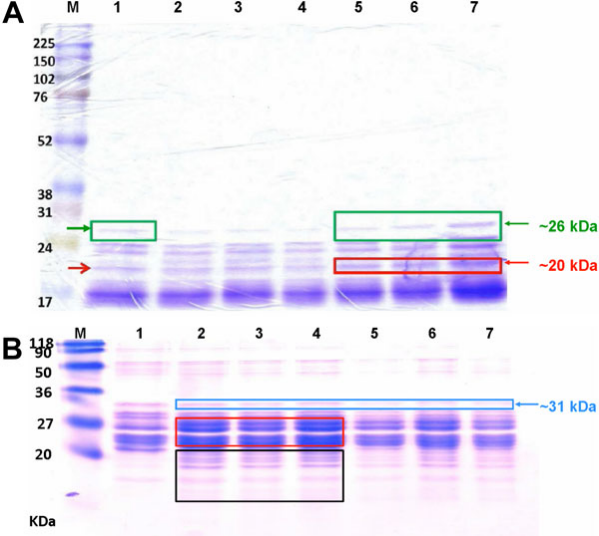
SDS–PAGE analysis of selenite-induced rat lenses treated with catalin equivalent to 2.5 and 5 mg/kg pirenoxine (PRX). M, marker; lane 1, normal; lane 2, control (Se only); lane 3, Se+catalin formualted vehicle; lane 4–5, Se+catalin equivalent to 2.5 mg/kg PRX; lane 6–7, Se+catalin equivalent to 5 mg/kg PRX. Duplicated samples from different lenses of rats pretreated by different catalin doses were analyzed. **A**: A 12.5% SDS–PAGE of soluble fractions of lenses and Amersham Rainbow Marker were used. Red arrow, 20-kDa proteins; red box, more preserved 20-kDa proteins in catalin-treated rats; green box, preserved 26-kDa proteins. **B**: A 20% SDS–PAGE of insoluble lens fractions and Fermentas SM0441 Marker were used. Blue box, 31 kDa proteins; red box, 21–25 kDa; black box, proteolytic fragments.

The protective effects of catalin solutions containing 0.016~0.1 μM PRX against selenite-induced precipitations were assayed. Percentages of the OD for groups incubated with the catalin-formulated vehicle were obtained by dividing by the ones for the selenite-only controls; the results were 88.3%~98.6%, as shown in Figure 4A. Turbidity formation was significantly delayed in groups incubated with catalin containing 0.032~0.1 μM PRX on incubation day 1, compared to the controls (p<0.05). The efficacy of catalin against selenite insults was further studied in the selenite-induced cataract rat model. The average cataract scores of the control (selenite only) and 5 mg/kg catalin-treated group on postinduction day 3 were 2.4±0.4 and 1.3±0.2, respectively (p<0.05), as shown in Figure 4B. There was no significant difference in opacity scores after post-induction day 4 in groups treated with catalin (2.5 and 5 mg/kg), compared to the controls. Results indicated that catalin may delay cataract formation. SDS–PAGE analysis (Figure 5A) showed catalin treatment decreased lens protein degradation in the fraction of water-soluble proteins; however, catalin at 5 mg/kg preserved 20- and 26-kDa proteins similar to normal lens proteins, particularly water-soluble ones (Figure 5A). In Figure 5B, fewer 31-kDa proteins were seen in selenite injected rat lenses and increased insoluble proteins in the 21–25 kDa. Catalin pretreatment slightly protected water-insoluble lens proteins.

## Discussion

Higher levels of calcium ions in human lenses with cortical cataracts indicate a possible role in opacification [40,41]. The increase in calcium ions in the cataract lenses of cataract animal models was correlated to proteolysis of lens crystallins [42-44]. This increase causes lens turbidity [38] by activating calpain, a protease present in the lens. Cataracts are well known to result from proteolysis of lens crystallins [42-44], lens crystallin aggregation [45], calcium-activated calpain-induced lens turbidity [15,46], and selenite-induced lens protein turbidity [13]. Both Wu et al. [15] and Chen et al. [47] reported that the ability to chelate calcium could be an important factor in preventing cataracts. Followed by our previous chemistry study for PRX [14], this study shows dose-dependent anti-cataract effect of PRX (0.03~0.3 μM; Figure 3) against selenite/calcium-induced turbidity formation. Catalin eye drop solution equivalent to 0.032–0.1 μM (Figure 4A) also ameliorated selenite-induced turbidity.

In addition to the above-mentioned factors, UV-induced oxidative damage is highly correlated with cataractogenesis [48,49]. Among five regions of solar radiation, UVC and UVB are responsible for photochemical reactions. This study is the first report to show that PRX up to 0.1 mM did not protect γ-crystallins [50], a fraction of lens proteins sensitive to oxidation, against UVB-induced photo-oxidation, but that PRX at 1 mM did protect γ-crystallins against UVC irradiation (Figure 1A). Here, we used the same wavelength (312 nm) of UVB as in the Ciuffi et al. [51] cornea study, but our assay system was more similar to a study of UVB-induced γ-crystallin turbidity [36]. Our negative results for PRX may have been due to the fact that our assay system used prolonged UVB exposure times of up to 2~6 h and accumulated 30 times the UVB insults (~24 J) of those used in the Ciuffi et al. [51] cornea study (up to 0.8 J). Therefore, doses of PRX (0.1~100 μm) used in the current study were insufficient to protect against UVB insults. Furthermore, the stability of PRX under UVB exposure is unclear. Pyridophenoxazine of PRX consists of two moieties of 3-hydroxykynurenin, a known photosensitizer which increases lens crystallin aggregation with UVB exposure [52]. PRX was thought to form 3-hydroxykynurenin after UVB irradiation, in turn enhancing protein aggregation; this was indeed observed in the current study.

Even though UVC can be blocked by the atmosphere or ozone layer, environmental changes can lead to increased exposure to UVC. The observed anti-UVC results in those incubations with catalin solutions containing PRX at up to ~100 μM indicated that CataV contains other ingredients which may also provide anti-UVC activity, resulting in extra photoprotection. However, since our catalin solution containing 100 μM PRX was prepared by dissolving catalin powder, the protective effect of a catalin solution containing 100 μM PRX appeared to have been due to CataV. The dose-dependent results shown in Figure 2B may have been due to the serial dilution of CataV. Therefore, the catalin eye drop solution provided anti-UVC protection due to PRX and other components. Similar to the results described in our earlier study of coumarins, UVC irradiation for 2~6 h resulted in increased turbidity and decreased protein bands at 21 kDa. The observed results may have been due to the insult of free radicals on γ-crystallins that further modified the protein structures [32]. The presence of 1 mM PRX protected γ-crystallins irradiated with UVC. The mechanism may be due to the radical-scavenging activity of PRX [53,54] against UVC-induced free radicals, singlet oxygen, and superoxides.

Concentrations of PRX and catalin which ameliorated lens protein turbidity formation were at micromolar levels, similar to those used in the Cantore et al. [55] cornea culture study; however, results in Figure 2 demonstrate that PRX, even up to 100 μM, did not inhibit calpain proteolysis. These results indicate that the mechanisms for this activity against selenite-induced turbidity may be only partially related to the chelating of calcium and selenite in 1: 3 and 1: 6 ratios, respectively, reported in our earlier study [14], because the chosen levels (0.03~100 μM) of PRX (Figure 3A,B and Figure 4A) were only trapping up to 0.09~600 μM calcium and selenite cations according to the reported chelation ratios, which are much smaller than the 10 mM of selenite/calcium used. This may account for the observed initial in vitro protective results, instead of sustained activity compared to CataV. An additional reason why 100 μM PRX did not inhibit calpain activation by 1.5 mM calcium was that PRX itself may have less affinity for calcium compared to calpain. In addition, PRX or components of catalin, unlike E64, might not be able to directly bind to calpain to inhibit calpain activity. Clarification regarding other mechanisms for protecting lens proteins against selenite-induced turbidity remains for future investigations.

The initial protective effects were further confirmed by the early protection from cataract formation following systemic catalin (5 mg/kg) given for 3 days before cataract induction on post-induction day 3 in a selenite-induced cataract rat model (Figure 4B). Such results are consistent with suggestion made by the majority of exponents of catalin, that catalin is useful only in an early stage of human cataract formation, but no medical therapy effect of catalin was reported from a clinical trial study by Angra et al. [56]. Our data using an intravitreal injection of a catalin solution (1 mg/ml equivalent to the clinically recommended maximum daily dose of 0.025 mg) following selenite injection also showed no significant differences in opacity scores of post-induction day 5 (data not shown). Therefore, catalin seems to be useful in cataract prevention, but not for therapy.

The initial beneficial effect of catalin not persisting for more than 4 days may have been due to catalin administration being discontinued after selenite induction. Inadequate doses of catalin (2.5~5 mg/kg) used in the current study might also account for the results, since a higher dose (20 mg/kg, intraperitoneally) of PRX, which inhibited alloxan-induced diabetic cataractogenesis, was given daily for 6 weeks [8]. A small protective effect of catalin at post-induction day 4 was also seen in the Ito et al. [57] study, in which 0.24 mg/ml of catalin (four instillations daily) was chosen. It was speculated that more-frequent systemic dosing following cataract induction by selenite may provide better protection from cataractogenesis.

Soluble fractions analyzed by SDS–PAGE after various treatments in vivo indicated that samples from rats treated with 5 mg/kg PRX were similar to normal ones. The more preserved in 20 and 26 kDa protein bands, suggested as αA-crystallins and β-crystallins, were seen in samples from individual rats with less severe cataract formation after catalin pretreatments, as reported in a lenticular protein profile of selenite-induced cataractogeneis [58]. The observed increase in new proteins, which ranged between 21 and 25 kDa and below 20 kDa (Figure 5B) in the insoluble fractions, may have been due to hydrolysis of 20, 26, and 27 kDa as reported during selenite cataractogeneis [42,59]. The loss of 31 kDa β-crystallin and the accumulation of polypeptides below 31 and 19 kDa of the insoluble fractions in rats with cataract development were similar to the results of Tamada et al. [60].

In conclusion, PRX protected crystallins against UVC-, selenite-, and calcium-induced lens protein turbidity; however, it might be detrimental under UVB exposure. Catalin may be useful in ameliorating UVC-induced turbidity formation, but not in inhibiting calpain-induced proteolysis of lens crystallins in vitro. The transient protection by catalin against selenite-induced turbidity of crystallin solutions in vitro was supported by the ameliorated cataract scores in the early stage of cataractogenesis in vivo by subcutaneously administered catalin. Modification of the catalin-formulated vehicle is recommended to improve its overall protective effects against the cataract-induction factors presented here. Further studies are warranted to assess the clinical efficacy and optimal doses of PRX of catalin to ameliorate various types of cataract development.
